# The Level of Free Fetal DNA as Precise Noninvasive Marker for Chromosomal Aneuploidies: First Results from BALTIC Region

**DOI:** 10.3390/medicina56110579

**Published:** 2020-10-30

**Authors:** Danielius Serapinas, Evelina Boreikaitė, Agnė Bartkevičiūtė, Kristina Norvilaitė, Andrius Narbekovas, Daiva Bartkevičienė

**Affiliations:** 1 Department of Family Medicine, Lithuanian University of Health Science, LT-50161 Kaunas, Lithuania; dserapinas@gmail.com; 2 Research Center on Marriage and Family, Vytautas Magnus University, LT-44260 Kaunas, Lithuania; eka.dr.gyvybe@gmail.com; 3 Clinic of Obstetrics and Gynecology, Institute of Clinical Medicine, Faculty of Medicine, Vilnius University, LT-01513 Vilnius, Lithuania; evelina.boreikaite@gmail.com (E.B.); kristina.norvilaite@gmail.com (K.N.); 4 Clinic of Infectious Diseases and Dermatovenerology, Institute of Clinical Medicine, Faculty of Medicine, Vilnius University, LT-01513 Vilnius, Lithuania; agnebartkeviciute@gmail.com

**Keywords:** non-invasive prenatal testing, bioethics, sensitivity, chromosomes

## Abstract

*Background and objectives:* Noninvasive prenatal testing (NIPT), which has been introduced clinically since 2011, uses the circulating cell-free fetal DNA in the maternal blood to evaluate the risk of a chromosomal anomaly. The aim of this study was to examine the effectiveness of NIPT using a single nucleotide polymorphism method. *Materials and Methods:* A retrospective study was conducted between 2013 and 2019. The Natera Panorama test was used to analyze the risk of trisomies 21, 18, 13, X monosomy, trisomy, and other sex chromosome abnormalities. A positive result of NIPT for aneuploidy was confirmed by invasive testing. *Results:* 850 women with a singleton pregnancy participated in the study. The median fetal fraction was 9.0%. The fetal fraction was lower in the no-call group (3.1%) compared with the group that received a call (9.1%) (*p* < 0.001). A positive correlation was determined between the gestational age and the fetal fraction (*r* = 0.180, *p* < 0.001). The overall positive predictive value (PPV) of NIPT for trisomy 21 (*n* = 9), trisomy 18 (*n* = 3) and XYY syndrome (*n* = 1) was 100%. *Conclusions:* The results of present study showed 100% PPV effectiveness of NIPT Panorama test detecting trisomies of 21 and 18 chromosomes, as well as XYY syndrome in the studied cohort. Therefore, NIPT due to its high PPV, significantly reduces the need for invasive testing, thereby reducing the risk of miscarriage and stillbirth.

## 1. Introduction

A non-invasive prenatal test (NIPT) was introduced in clinical practice in 2012. A cell-free DNA (cfDNA) is isolated from maternal blood and analyzed using three different methods: single nucleotide polymorphisms (SNPs), massively parallel sequencing of the whole genome (MPS), and targeted sequencing [1]. Plasma cell free DNA consists of maternal and fetal genomic DNA fragments [2]. The majority of cell-free fetal DNA (cffDNA) is derived from apoptotic placental trophoblastic cells [3] and the remainder is derived from apoptotic hematopoietic stem cells [4]. The amount of fetal fraction is an important factor for the accuracy of the test [5]. In accordance with the meta-analysis data, between 0.5% and 6.1% of all the tests fail to deliver results because of a low fetal fraction [6]. A cffDNA can been found in maternal blood as early as five weeks of gestation [7], but DNA levels at that time are still too low to be tested. The Natera Panorama test, which is based on the single nucleotide polymorphisms technology, could provide accurate results when the fetal fraction is ≥ 2.8%. Consequently, the amount of cffDNA in maternal blood is sufficient to perform this test accurately from gestation week 9–10 [8]. However, the test is considered to be related to failures when the fetal fraction is ≤3–4% depending on different laboratories [9].

NIPT can be used as a primary screening test or a further testing option rather than other standard methods deployed to identify an increased risk [10]. One of the major benefits of NIPT is the ability to ensure high quality. This test is able to confirm trisomy 21 with the detection rate of 99% and false positive results—of less than 0.1% [6]. The positive predictive value (PPV) for trisomy 18 and trisomy 13 are 97.7% and 96.1%, respectively, while the false-positive results are 0.04% and 0.06%, respectively [6,11,12,13]. The detection rate of sex chromosome abnormalities is lower than in other trisomies, but still remains high: monosomy X—90.3% [6]; 47, XXX, 47, XXY, 47, XYY—93.0% [14,15,16]. NIPT allows avoiding complications that are associated with the invasive diagnostic procedures [17]. It is important to note that patients should be informed during each prenatal consultation that NIPT is only a screening method. The cases of high risk must be confirmed by the invasive prenatal testing (amniocentesis or chorionic villus sampling) [18] which also provides information on the fetal chromosomal rearrangements (translocations, inversions, duplications, deletions) and other non-chromosomal abnormalities.

The objective of this study was to evaluate the maternal and fetal characteristics that can correlate with the concentration of cffDNA and to investigate the relationship between the different chromosomal abnormalities (trisomies of 21, 18, 13 chromosomes; triploidy, sex chromosome abnormalities, such as X monosomy, XXX, XXY and XYY) and fetal fraction. We aimed to examine the PPV of a single nucleotide polymorphisms-based NIPT in the Lithuanian female population.

## 2. Materials and Methods

We collected the data retrospectively on pregnant women with a singleton pregnancy who underwent NIPT in InMedica clinic during 2014–2019. The exclusion criteria were multiple gestation and gestational age ≥21 weeks. NIPT was performed in the following groups of women: (1) aged 35 years or older with mean age 37.7 years (range 35–49 years); (2) with a high risk identified after the first-trimester screening for chromosomal abnormalities years or older with mean age 34.1 years (range 23–42 years); (3) without the increased risk with mean age 28.5 years (range 19–32 years). NIPT was repeated after the first test was uninformative. The main reason why the laboratory could not deliver the result was the fetal fraction below the threshold ≤2.8%. The study was authorized by the Ethics Committee of the Lithuanian University of Health Sciences, and a written informed consent was obtained from all participants.

The Panorama Test (NIPT) was performed on all the subjects of our study. The medical personnel took two blood samples of 10 mL from each subject. All the blood samples were transported within 48 h by plane to the Natera laboratory in San Carlos, CA (USA). The samples were analyzed as previously described using validated methodologies for cfDNA isolation, polymerase chain reaction amplification targeting 19,488 SNPs, high-throughput sequencing, and the analysis with the next-generation aneuploidy test using the SNPs (NATUS) algorithm [15,19,20].

The Natera Panorama test was used to analyze cffDNA from maternal blood for the detection of the following chromosomal abnormalities: (1) trisomies of 21, 18, 13; (2) X monosomy; (3) triploidy; (4) other sex chromosome abnormalities and fetal gender. All the samples with a risk score ≥9:10 were reported as a high risk for fetal aneuploidy and the samples with the risk scores <1:10 000 were considered of low risk. The samples were processed and the results were obtained within 5–7 business days. The high-risk results were confirmed by the invasive diagnostic procedures (amniocentesis or chorionic villus sampling). A follow-up was performed by a telephone call for a low-risk group in order to ascertain that the infants would be born without chromosomal abnormalities.

The descriptive data of demographic information are presented as median and minimum/maximum values. Continuous variables for normal distribution were inspected using the Kolmogorov–Smirnov test. We used the Spearman and partial correlation tests to evaluate the correlation of various factors associated with fetal fraction. The Mann–Whitney U test was used for comparing the continuous variables between the groups. PPV was calculated by the formula PPV = true positive (TP)/(true positive (TP) + false positive (FP)). A *p* value < 0.05 was considered statistically significant. A statistical analysis was performed using the SPSS 23.0 program.

## 3. Results

**Figure 1:**
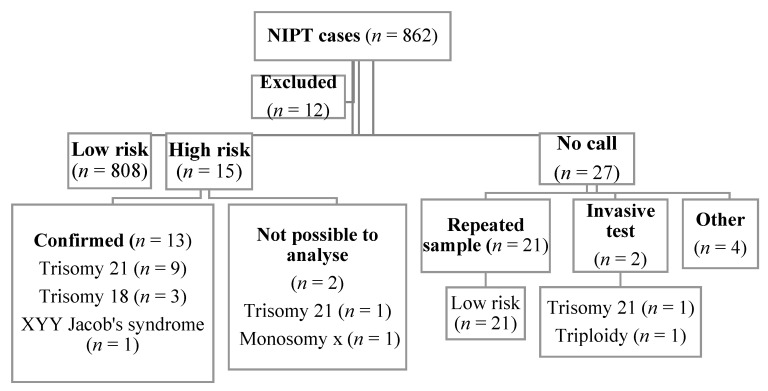
Flow chart of the study. NIPT-Noninvasive prenatal testing.

The flow chart for the study is presented in Figure 1. We collected the data of 862 women who received NIPT; the sample collection dates ranged between November 2013 and June 2019. Overall, after excluding 12 records, our study included 850 subjects.

The baseline characteristics of the whole study population are listed in Table 1. The median age of 850 cases was 35 years (range 19–49 years). More than half of women 58.1% (494/850) were ≥35 years old. Most of the blood samples, 79.8% (678/850), were taken in the first trimester of pregnancy and 20.2% (172/850) were taken during the second trimester of pregnancy.

**Figure 2:**
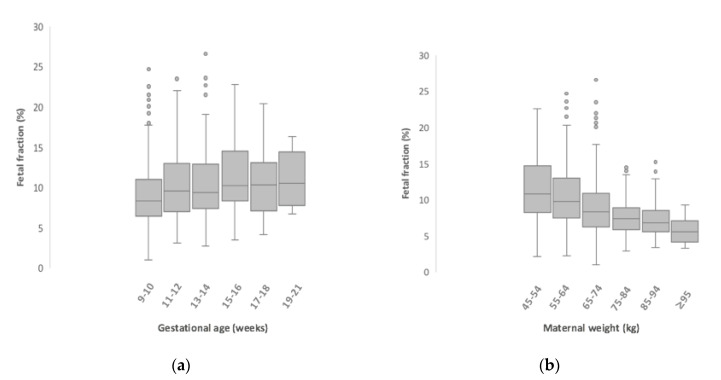
Relationship between the fetal fraction and the gestational age, (**a**) the fetal fraction and the maternal weight (**b**). Boxplot description: The top line of the box is 75th percentile. The horizontal line inside each box is the median. The bottom line of the box is 25th percentile. The vertical lines out of the box represent the minimum and maximum values. The circle outside of the box are outliers.

Of all 850 cases, the median fetal fraction was 9.0% (in the range of 1 to 26.6%). The fetal fraction of female fetuses was significantly higher than male fetuses (9.4% vs. 8.8%, *r* = −0.13, *p* < 0.001). We found a positive correlation between the fetal fraction and the gestational age (*r* = 0.180, *p* < 0.001) (Figure 2). Furthermore, a significant negative correlation between the fetal fraction and the maternal weight was revealed (*r* = −0.330, *p* < 0.001). The fetal fraction was lowest (5.6%) in the women’s group weighing ≥95 kg, while the highest fraction (11.1%) was estimated in the group weighing 45–54 kg. There is no correlation between the fetal fraction and the maternal age (*p* = 0.200).

The prevalence of high-risk cases among the study participants was 1.8% (15/850), including 1.2% (10/850) trisomy 21, 0.4% (3/850) trisomy 18, 0.1% (1/850) monosomy x and 0.1% (1/850) XYY (Jacob’s syndrome). The cases of trisomy 13 and triploidy were not detected in our study (the prevalence of these chromosomal abnormalities was 0%). The median fetal fraction of trisomy 21 and trisomy 18 cases was 9.8% (in the range of 4.8 to 20.0%) and 7.4% (in the range of 5.7 to 10.7%), respectively. The fetal fraction of a low risk group did not differ significantly from trisomy 18 (*p* = 0.245) and trisomy 21 (*p* = 0.514) groups.

We confirmed 13 (87.0%) of 15 high-risk cases: 12 cases using the invasive prenatal testing and a single case of XYY syndrome was confirmed using a biopsy of the fetal liver after miscarriage. One woman at a high risk of trisomy 21 refused to take the invasive test and terminated pregnancy. Monosomy X could not be confirmed due to the premature miscarriage. There were no false positive results in the group of subjects where the invasive test was successfully performed, so the positive predictive value of trisomy 21, trisomy 18, and XYY syndrome was 100% (Table 2).

NIPT was not informative in 3.2% of the samples (27/850). This was defined as a “no-call” group. The subjects were included in this group when the first test was non-reportable. The laboratory could not analyze the fetal fraction in 10 of 850 cases (1.2%), while in 7 cases (0.8%) the fraction was less than the limit value <2.8%. NIPT was repeated in 77.8% of women (21/27) (in two women the test was performed three times) and it showed a low risk. NIPT was not repeated in 6 out of 850 participants (0.7%), instead, for two women 7.4% (2/27) the invasive tests were performed which showed a high risk for trisomy 21 and triploidy (Table 3). The median fetal fraction in the no-call group (3.1%) was significantly lower than in the group that received a call (9.1%) (*p* < 0.001). The lowest maternal weight was found in the no-call group, but it did not differ significantly compared with the group that received a call (63.0 kg vs. 67.0 kg, *p* = 0.166).

## 4. Discussion

This is the first study in the Baltic States that has analyzed the PPV of NIPT and examined the factors that affect the fetal fraction levels. The overall PPV in detecting trisomies 21, 18, and XYY syndrome in our subjects was 100%. To the best of our knowledge, this is the only study with no false positive cases. Of course, all NIPT tests including Natera Panorama generally have some amount of false positives, but in our case it could be not yet observed because of a relatively low study cohort for epidemiological studies. Furthermore, increasing the numbers of participants really could cause more cases of false positives to appear. Previous publications showed that PPV was 97.4% for trisomy 21 and 88.9% for trisomy 18 [21]. However, an Indian study estimated PPV of trisomy 21 and sex chromosome abnormalities XXX, XXY (80% and 50%, respectively), which was much lower compared with our results, but PPV of trisomy 18 was identical to the findings obtained from our research (100%) [22]. An important issue is that any high-risk score in NIPT should be confirmed by invasive prenatal diagnosis before any decisions about pregnancy. While reducing the invasive testing rates, despite the low complication rates of these tests, NIPT saves the lives of some fetuses who could potentially be miscarried as a result of the diagnostic testing with CVS or amniocentesis.

In the light of the previous studies, our findings support the results obtained by other scholars that the higher maternal body weight is associated with the lower fetal fraction [23]. In the highest weight group (≥95 kg), the fetal fraction was twice as low compared to the lowest weight group (45–54 kg). Ashoor et al. also found an inverse relationship between the fetal fraction and the maternal body weight [24]. Other authors have shown an increase of no-call cases in the group of overweight and obese mothers compared to the normal weight group [25]. In our study, the subjects of the group of no-call cases had a higher body weight than the rest of the subjects under analysis, however, no statistically significant relationship was observed between them. Several theories have been proposed to explain the relationship between the body weight and the fetal fraction. One of them claims that remodeling of adipose tissue in obese pregnant women is associated with higher levels of the total circulating cell-free DNA. The fetal DNA level is constant, but the increase of maternal DNA leads to an overall lower fetal fraction [26]. Another theory explains that pregnant women with a high weight have lower cffDNA because of the dilution effect from an increased plasma volume [27].

Similar to many other authors, a direct correlation between the fetal fraction and the gestational age was found in our study [23,28]. According to Wang et al., the fetal fraction increased by 0.10% per week between 10 and 21 weeks of gestation, and starting from 21st week, by 1% per week [29]. However, there was one study that found no association between the fetal fraction and the gestational age [30].

Our study is the only one demonstrating that the fetal fraction of female fetuses was significantly higher than that in male fetuses. There are a few authors who have examined the impact of the fetal sex on the fetal fraction, but no significant difference was found [24,28]. Further studies with a larger study population are needed to support our findings.

The percentage of no-call cases ranges from 1% to 5% in many studies [31]. No-call cases accounted for 3.2% of all our study population, and it was consistent with the results found by Verma et al. (2.3%) [22]. We found that the median fetal fraction in the group of no-call cases was more than three times lower in comparison with other groups. Thus, our study also contributes to the conclusions that the fetal fraction that is too low is one of the major factors affecting non-informative NIPT [32]. The NIPT results on redraw were with success rate of 77.8%, while Gray et al. reported redraw success rate of 60% [33]. It is known that the risk of aneuploidy is much higher in no-call cases, reaching 2.7–23.0% [34]. In our study, out of six cases when NIPT was not repeated, 7.4% cases (trisomy 21 and triploidy) were confirmed by invasive methods.

The amount of the fetal fraction is essential in aneuploidy detection by NIPT, therefore, different chromosome aneuploidies could have an impact on the accuracy of NIPT [35]. The detection of trisomy 21 is the most accurate of all the chromosomal abnormalities due to the significantly higher fetal fraction compared to the low-risk group, but our results did not support this [24]. Additionally, our data showed that the fetal fraction of the trisomy 18 group did not differ significantly from the low-risk group. Nevertheless, the fetal fraction of trisomy 18 group was lower compared to the low-risk group in the Suzumori et al. study [36].

Several limitations of this study should be considered that include a low sample size and few subjects in risk groups. We have not evaluated the influence of obesity on the fetal fraction due to the lack of data on the female height. Furthermore, the risk for some women could not be confirmed because of miscarriage and the decision to refuse the invasive prenatal testing. Moreover, our study lacks the follow-up data on the possible chromosomal abnormalities in newborns whose mothers were included in the low risk group.

## 5. Conclusions

Our study has showed that all NIPT high-risk cases (9 of trisomy 21, 3 of trisomy 18, and 1 of XYY syndrome) were confirmed with invasive testing. It reveals that the NIPT Panorama test in the studied cohort has 100% PPV value. NIPT is likely to be used in prenatal screening at the first trimester due to its high PPV. However, NIPT could not replace the first trimester fetal ultrasound scan, which provides the clinicians with comprehensive information about other fetal structural defects and congenital anomalies.

## Figures and Tables

**Table 1:** Maternal and fetal characteristics of the study population.

	*n*	Median (Min–Max)
**Maternal age (in years)**		
All	850	35 (19–49)
Low risk	808	34 (19–49)
High risk	15	38 (29–47)
No call	27	35 (27–42)
**Gestational age (in weeks)**		
All	850	11 (9–21)
Low risk	808	11 (9–21)
High risk	15	11 (9–18)
No call	27	10 (9–16)
**Maternal weight (kg)**		
All	850	62.9 (44.6–174.8)
Low risk	808	63.0 (44.6–174.8)
High risk	15	63.1 (51.0–90.1)
No call	27	67.0 (51.8–94.3)
**Fetal fraction (%)**		
All	840	9.0 (1–26.6)
Low risk	808	9.2 (2.9–26.6)
High risk	15	6.4 (4.8–20.0)
No call	17	3.1 (1–5.9)

**Table 2:** Positive predictive values of non-invasive prenatal screening.

	*n*	Confirmatory Test	FP	FF (%)	PPV
**High risk**	15	13	0	6.4	100%
• **Trisomy 21**	10	9	0	9.8	100%
• **Trisomy 18**	3	3	0	7.4	100%
• **Monosomy x**	1	0		8.4	
• **XYY (Jacob’s syndrome)**	1	1	0	6.4	100%

FP-false positive, FF-fetal fraction, PPV- positive predictive value.

**Table 3:** No-call cases.

	*n* (%)	Risk
**Repeated samples**	21 (77.8%)	Low risk
**Invasive tests**	2 (7.4%)	Trisomy 21
		Triploidy
**Miscarriage**	2 (7.4%)	-
**Refused to repeat a sample**	2 (7.4%)	-
**Total**	27	
